# Resin Infiltration in Dental Fluorosis Treatment—1-Year Follow-Up

**DOI:** 10.3390/medicina57010022

**Published:** 2020-12-29

**Authors:** Francesca Zotti, Luca Albertini, Nicolò Tomizioli, Giorgia Capocasale, Massimo Albanese

**Affiliations:** Section of Dentistry and Maxillofacial Surgery, Department of Surgical Sciences, Paediatrics and Gynecology, University of Verona, 37134 Verona, Italy; francesca.zotti@univr.it (F.Z.); lucalbe93@gmail.com (L.A.); nicolo.tomizioli@studenti.univr.it (N.T.); massimo.albanese@univr.it (M.A.)

**Keywords:** dental fluorosis, resin Infiltration, dental Materials, minimally technique

## Abstract

*Background and Objective:* Dental fluorosis is a disease affecting dental hard tissues featured with white or yellowish lesions. Several treatments are proposed in the literature, some even invasive. This clinical study aimed to evaluate the effectiveness of resin infiltration in terms of lesions resolution, trend of sensitive teeth and satisfaction of patients over time. *Methods and Material:* 200 fluorosis lesions were treated using ICON infiltrating resin (DMG, Hamburg, Germany). Parameters related to patients were collected by a questionnaire and analyzed aesthetic dissatisfaction about lesions, Shiff Air Index Sensitive Scale, sensitive teeth after treatment, the satisfaction of duration of treatment. The same operator measured dimensions of lesions Tooth Surface Index of Fluorosis (TSIF) and numbers of etching cycles needed for treating lesions. Statistical analysis was performed. The follow-up was of 1-year a measurement were performed at baseline (t0), immediately after the treatment (t1) and every three months during the observation period. *Results:* All lesions disappeared after one treatment. Pain or sensitive teeth were reported inside the 72 h and they disappeared after. Statistical analysis showed highly statistically correlation between etching cycles and the dimension of lesions and TSIF at the time-points evaluated as well as for pain during treatment, whereas a statistical significance was not noticed where etching cycles were correlated to sensitive teeth after 72 h. Overall, the treatment was found to be statistically significantly associated with differences in answers of aesthetic dissatisfaction between t0 and t1 and those collected between t1 and t2. Between t2 and t3 and between t3 and t4 no statistical differences were found in answers of patients about dissatisfaction, indicating the stability of the results. *Conclusions:* The ICON resin infiltration technique was found to be effective in lesions resolution with steady results.

## 1. Introduction

Dental fluorosis is a disease caused by excessive deposition of fluoride in enamel featured with white or yellowish lesions on tooth surface [1]. This condition affects both function and aesthetics and it mostly occurs in the population residing in areas with a considerable amount of fluoride in tap water since the pediatric age [2]. The childhood, further, contributes increasing the amount of intake of fluoride due to the inability of pediatric patients not to ingest toothpaste containing fluoride [2,3].

Tooth development could be negatively affected by high doses of fluoride, especially during the mineralization of the enamel tissue; indeed the increase of fluoride causes a decrease of free calcium ion concentration in mineralizing matrix process with a consequent delay of degradation of matrix proteins. Also, the presence of fluoride provokes a defective crystal growth responsible for typical features of fluorosis s [1,4].

Besides, fluoride is an essential member in the prevention of dental caries, actually, at recommended levels; it is responsible for hardening enamel tissue and increasing the strength of hard tissues against the acid and demineralizing attacks in the oral cavity. Moreover, accurate and appropriate use of fluoride is found to be effective in re-mineralizing white spots or demineralized lesions [5].

Recommended intake of fluoride for primary prevention of caries is reported to be 0.05 to 0.07 mg F/Kg/day [6] and, when concentration is more than 1.5 to 4 mg/L as the World Health Organization (WHO) recommends, dental fluorosis occurs [7]. In this perspective, an important issue is to prevent the abnormal intake of fluoride in children and it has to be advised by parents and health care figures by paying attention to dental hygiene habits [8], to feeding and tap water drinking.

Features of dental fluorosis are various and the extent of it could be clinically heterogeneous: spotted enamel, brownish or yellowish lesions, pitted surfaces and thin and horizontals striations can appear on all tooth surfaces affecting also dentin [9,10]. Surely, more evident lesions could affect self-esteem and are remarkable in terms of discomfort, especially in younger patients [11].

Literature reports several ways to clinically assess dental fluorosis. The Tooth Surface Index of Fluorosis (TSIF) was introduced in 1984 and is commonly used to assess the disease. A score is given to all surfaces of teeth and it allows to distinguish between different degrees of pitting, staining and staining with pitting [12].

Several treatment strategies were proposed for dental fluorosis, they depend on the severity and extent of the disease. The most frequently reported are micro and/or macro-abrasion, dental bleaching, composite restorations, veneers and prosthetic crowns; moreover, a recent review indicated the resin infiltration technique as the most performing and promising approach [13]. This meets the concept of minimally-invasive restorative dentistry and allows to reach satisfying results avoiding unneeded tissue removal.

The penetration of the resin into the fluorosis lesion is made possible thanks to etching the enamel surface performed with 15% solution of hydrochloric acid for 120 s. This procedure determines the elimination of a 58 µm (37 µm) surface layer of enamel, making accessible the hypomineralized site. Thus, a low-viscosity resin penetrates the porous enamel [14,15]. 

The improvement of aesthetics using resin infiltration is due to modification of refracting index (RI) from porous lesion composed by air (RI = 1) and water (RI = 1.33) to the surface infiltrated by resin (RI = 1.42–1.44) closer than healthy enamel (RI = 1.62–1.63), therefore a restoration with enamel-like optical characteristics is obtained [16]. 

However, the deeper lesions or those already featured with pits and cavitated fissures could require more extended treatment. 

This clinical study aimed to evaluate the effectiveness of resin infiltration technique in terms of lesion resolution, the trend of sensitive teeth and satisfaction of patients affected by dental fluorosis over time. 

## 2. Materials and Methods 

This study was carried out at the Department of Surgical Sciences, Pediatrics and Gynecology of the University of Verona from April 2019 to July 2020. 

All patients with permanent teeth, vestibular not pitted fluorotic lesions were treated and periodically recalled for dental check-ups and professional hygiene at our department. 

Exclusion criteria were extended to subjects with alternative food habits, such as vegan, suffering from eating disorders and assuming corticosteroids therapy [5,17,18,19,20] or not willing to be involved. 

All patients signed a written informed consent to take part in the study and to undergo treatment of resin infiltration (ICON, DMG, Hamburg, Germany). The consent of minor patients was provided by both parents. No further Ethics Committee was needed for this clinical study because this procedure is part of the clinical routine of Section of Dentistry and Maxillo-facial Surgery of the Department of Surgical Sciences, Pediatrics and Gynecology of the University of Verona. The study was conducted in accordance with the Declaration of Helsinki and the protocol was approved by the Review Board of the Section of Dentistry and Maxillo-facial Surgery, University of Verona. No ethical committee was required for this study because routinely already approved procedures were carried out.

**Figure 1:**
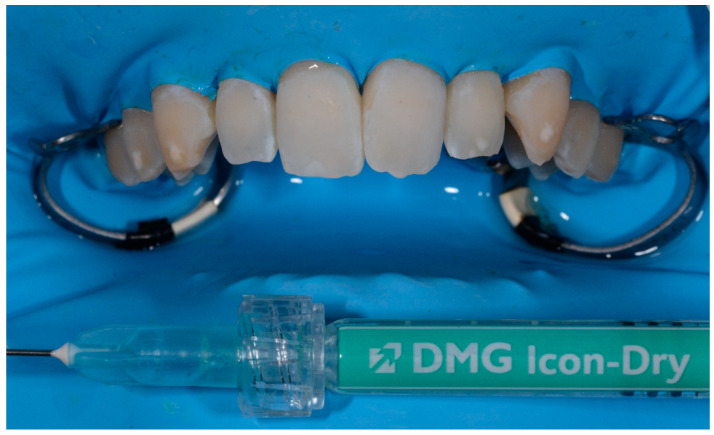
Application of ethanol.

**Figure 2:**
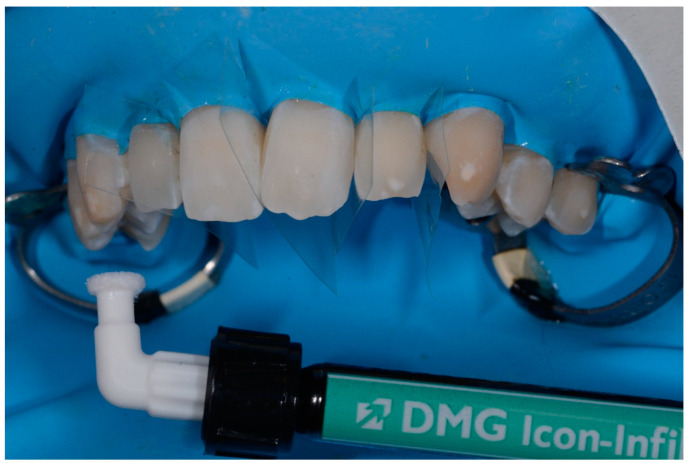
Application of infiltrating resin.

**Figure 3:**
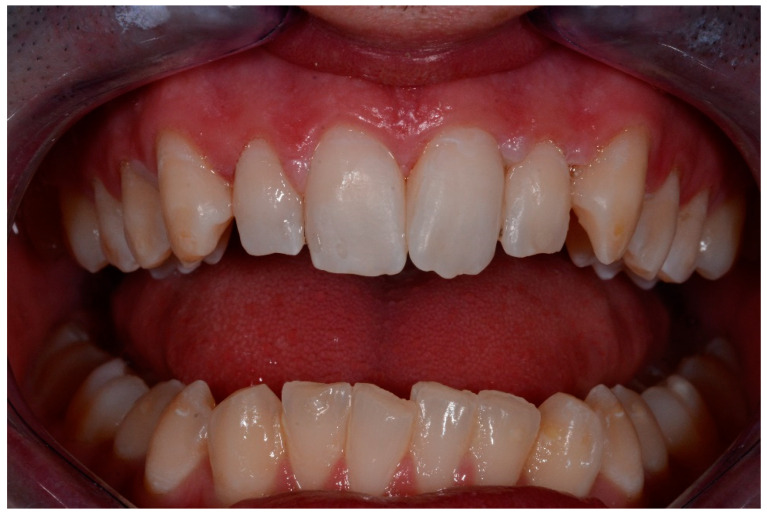
Finishing and final results.

All defects were managed by the same trained operator following instructions of the manufacturer (DMG, Hamburg, Germany):

- isolation with dental dam, hooks and accessories devices (dental floss, wedges, transparent matrices) where needed;
- cleaning of dental surfaces with a prophylaxis paste without fluoride;
- etching of lesions with 15% hydrochloric acid (Icon etch, DMG) for 120 s;
- rinsing of surfaces with abundant water for 30 s and drying with air jet;
- application of 99% pure ethanol (Icon-Dry, DMG) for 30 s; (Figure 1)
- assessment of needing for further etching cycles by the operator
- completed the drying of surfaces, application of infiltrating resin (Icon Infiltrant; DMG) left in position for 3 min; (Figure 2)
- elimination of excess material using cotton pellets in vestibular portion and dental floss in interproximal areas, thus photopolymerization with UV lamp for 40 s;
- second infiltration of resin, left in position for 1 min and polymerized for 40 s
- polishing of surfaces, check of contact points and finishing with abrasive stripes in interproximal areas requiring. (Figure 3)

Patient’s parameters were collected by administrating a questionnaire to patients as follows:

(a) Aesthetic dissatisfaction for lesions (VAS scale, 0: indifferent, 10: highly unsatisfied)
(b) Shiff Air Index Sensitive Scale (after treatment) [21]
(c) Sensitive teeth reported (after 72 h)
(d) Satisfaction of the duration of treatment (VAS scale, 0: not satisfied, 10: highly satisfied)
(e) Pain during treatment (VAS scale)

Lesion’s parameters were analyzed as follows:

- TSIF
- The main dimension of lesions (mm)
- Etching cycles needed

All parameters and measurements were performed by the same trained operator.

In order to measure the main dimension of lesions, an intraoral photographs of teeth affected was taken placing a periodontal probe near to lesion on the tooth to obtain proportions. 

Photographs were taken with D700 (Nikon, Tokyo, Japan) with Nikon AF-D DC 105 mm f/2 lens (Nikon, Tokyo, Japan) and R1C1 dual flash (Nikon, Tokyo, Japan) positioned on a camera tripod at 30 cm from the patient’s teeth. Camera was perpendicularly stabilized to the vestibular surface of the upper central incisors and setting used were the same for the pre- and postoperative pictures (f29, 1/60) [22].

Effective measurement of main length of lesions was determined by a digital software elaboration (Rasband, W.S., ImageJ, U. S. National Institutes of Health, Bethesda, Maryland, USA) of photographs [22] by the same operator. Measurements were taken twice for each lesion with a ten min interval in between. A single-measure interclass correlation coefficient (ICC) to evaluate the repeatability of these measurements was performed. ICC values change from 0 to 1. 0.01 indicates “poor” agreement; from 0.01 to 0.20 indicates “slight” agreement; from 0.21 to 0.40 indicates “fair” agreement; from 0.41 to 0.60 indicate “moderate” agreement; from 0.61 to 0.80 indicate “substantial” agreement; from 0.81 to 1.00 indicates “almost perfect” agreement; and 1 indicates perfect agreement.

**Figure 4:**
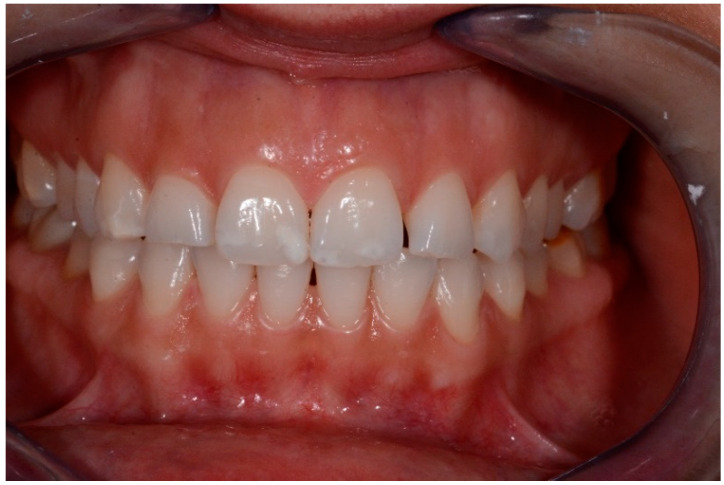
Baseline lesions.

**Figure 5:**
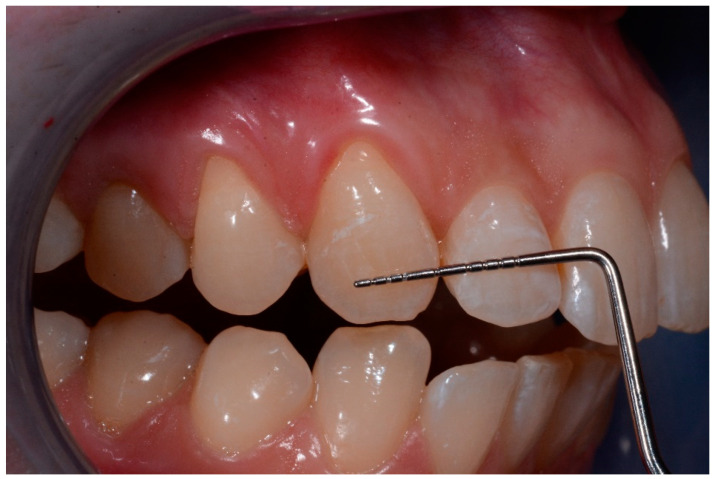
Baseline lesion with the periodontal probe.

**Figure 6:**
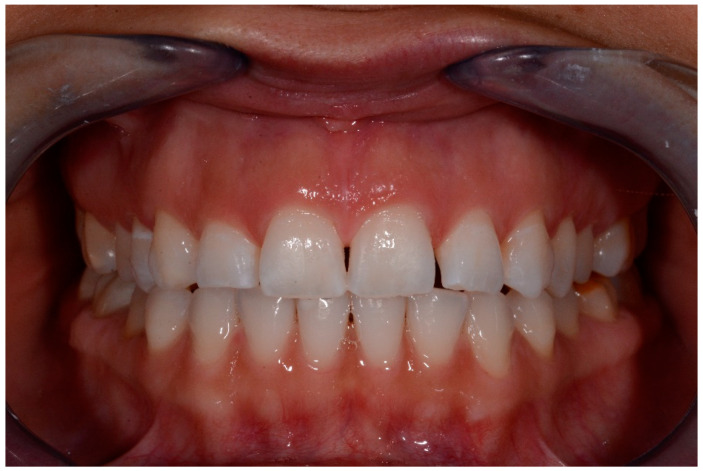
t1 lesions.

**Figure 7:**
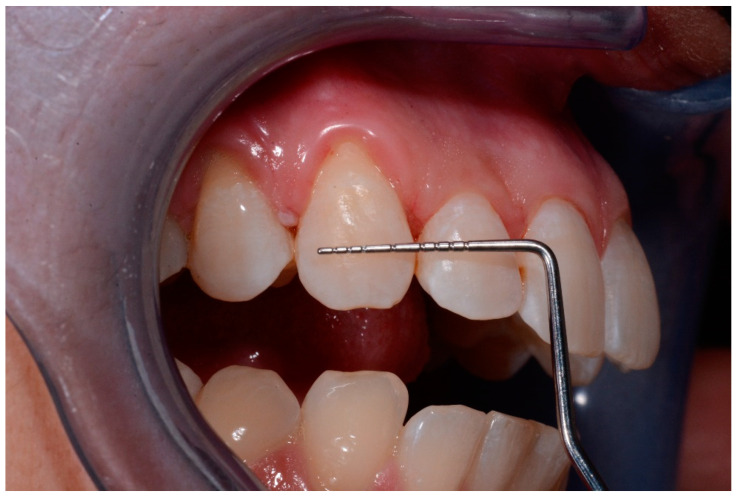
t1 lesion with the periodontal probe.

**Figure 8:**
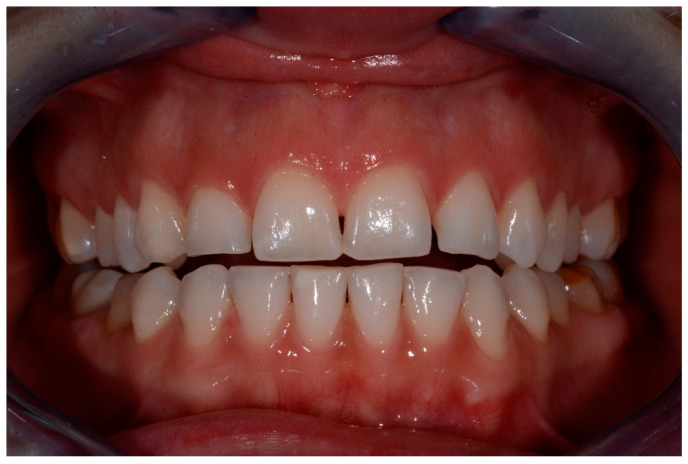
t2 lesions.

**Figure 9:**
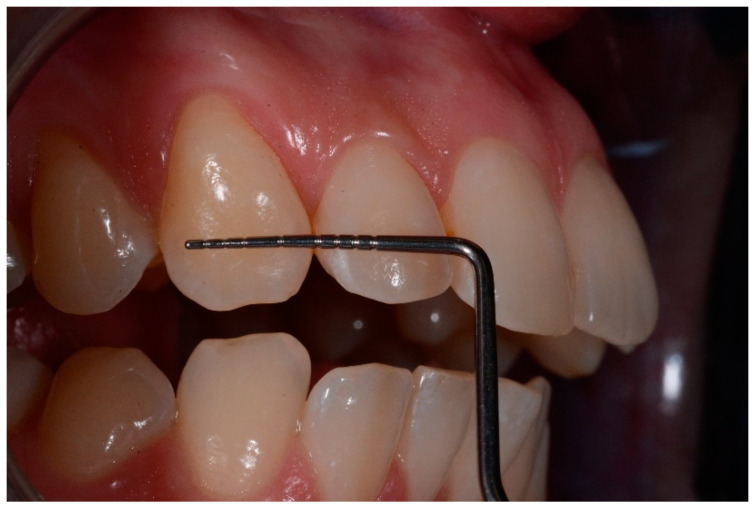
t2 lesion with the periodontal probe.

Intraoral photographs and parameters were taken at different time-points:

- t0: before the treatment (Figure 4 and Figure 5)
- t1: after treatment (Figure 6 and Figure 7)
- every 3 months during the observation period (t2, t3, t4) (Figure 8 and Figure 9)

### Statistical Analysis

Descriptive statistics were performed to evaluate data collected.

Etching cycles required were correlated by Spearman’s correlation with:

- Dimension of lesions at t0 and t1
- TSIF at t0 and t1
- Dental sensitivity values after 72 h of treatment
- Pain during treatment

Wilcoxon signed-rank test was performed to analyze:

- Aesthetic dissatisfaction for lesions at t0 and t1
- Aesthetic dissatisfaction for lesions at t1 and t2
- Aesthetic dissatisfaction for lesions at t2 and t3
- Aesthetic dissatisfaction for lesions at t3 and t4 (values in t4 were collected on 26 patients)

U Mann-Whitney test was performed to evaluate:

- Differences between aesthetic dissatisfaction of smokers and non-smokers patients (at t0 and t1)

Statistical tests were considered significant for *p* ≤ 0.05.

All statistical analyses were performed using Statistical Package for Social Sciences Version 25.0 (SPSS Inc., IBM, Chicago, IL, USA).

## 3. Results

A total of 200 dental fluorosis lesions were treated on 30 patients. Demographic characteristics of subjects involved are shown in Table 1.

**Figure 10:**
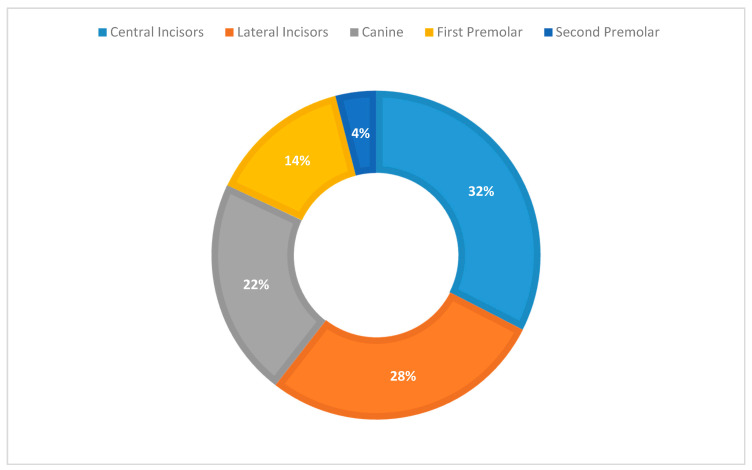
Distribution of lesions among teeth.

Figure 10 shows the distribution of lesions among different teeth.

The follow-up was of 1 year.

Frequencies of aesthetic dissatisfaction at t0 were scored 4 in the 3.3% of cases, 5 and 6 in 13.3%, 8 and 9 in the 16.6% of cases, 7 in the 26.7% and 10 in the 10% of cases. The aesthetic dissatisfaction at t1 was scored 0 in the 60% of cases, 1 and 2 in the 16.7 % of cases and 3 and 4 in the 3.3%. At t2, values of aesthetic dissatisfaction were reported as 0 in the 83.3% of cases, 1 in the 13.3% and 2 in the 3.3%. Both at t3 and t4, aesthetic dissatisfaction was scored 0 in the 100% of cases.

Details of Aesthetic dissatisfaction at timepoints (VAS scale values) were reported in Table 2.

**Figure 11:**
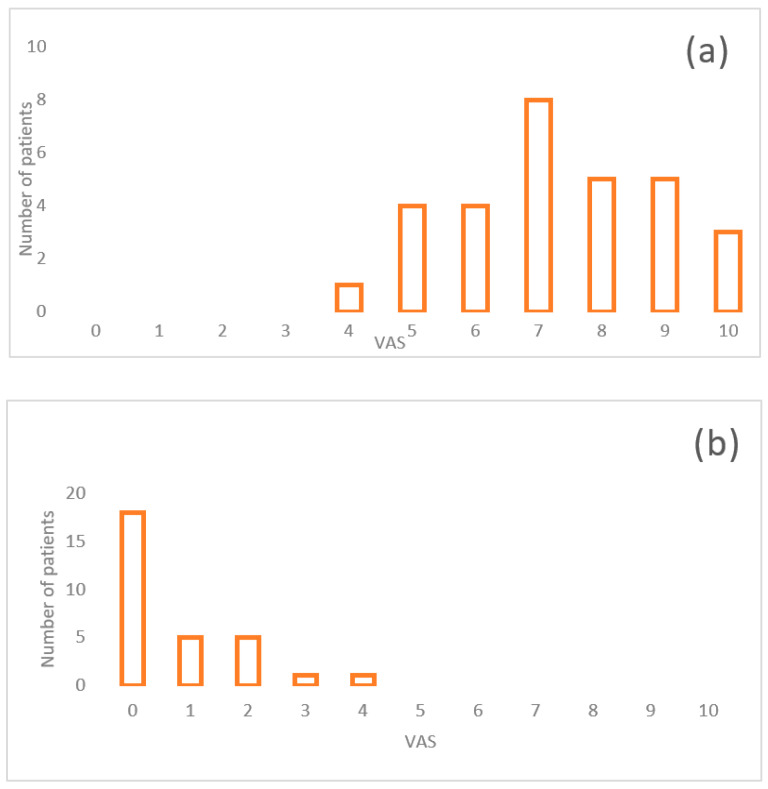

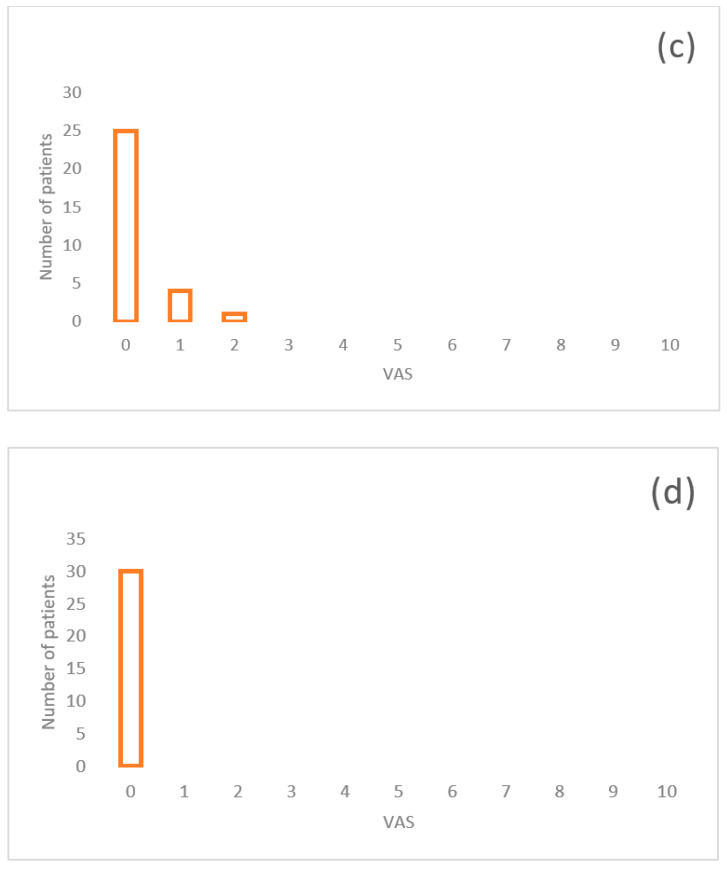
Aesthetic dissatisfaction (VAS) of patients at different time-points: (**a**) t0; (**b**) t1; (**c**) t2; (**d**) t3.

Figure 11 shows the dissatisfaction of patients at different time-points.

Shiff Air Index Sensitive Scale, indicating sensitivity during the treatment, was 0 for all patients at all time-points, except for one patient which reported a value of 3 at all follow-ups, due to periodontal affection.

Nineteen patients reported to not have sensitive teeth after treatment (0 value), only 11 patients reported sensitivity (2 value) disappeared within 72 h.

All patients answered to be satisfied of the duration of treatment. Satisfaction was scored as follows: 0 in the 3.3% of cases, 6 in the 6.7%, 7 in the 10%, 8 in the 30%, 9 in the 23.3% and 10 in the 26.7% of cases.

Concerning to pain during treatment, interviewed answered as follows: 0 in the 56.7% of cases, 1 in the 26.7%, 2 in the 10% and 4 in the 6.7% of cases.

The TSIF recorded at t0 were 1 (65.50% of cases), 2 (30.50%), 3 (4.00%), immediately after treatment TSIF was 0 (83.00% of cases), 1 (16.00%), 2 (1.00%).

Mean values for dimensions of lesions (mm+/− SD) were 2.66 mm (1.42) at t0 and 0.32 mm (0.88) at t1. Interclass correlation coefficient (ICC) value was found to be 1 for measurements.

**Figure 12:**
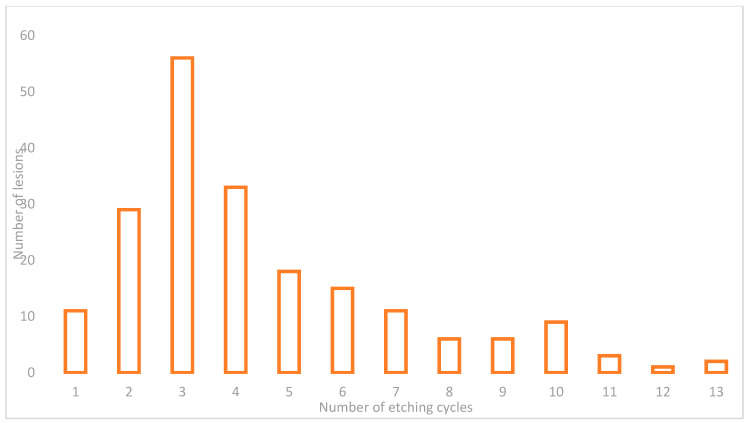
Number of etching cycles performed for each lesion.

Etching cycles needed were ranged between 1 to 13, with the majority of lesions (86%, not gauss-like distribution) having needed 1–7 etching cycles and only a few lesions needed a high number of etching cycles; however, in all case, enamel depletion was noticed. Figure 12 shows the etching cycles performed for each lesion.

Spearman’s correlation was found to be highly statistically significant for etching cycles and the dimension of lesions and TSIF at the time-points evaluated as well as for pain during treatment, whereas a statistical significance was not noticed where etching cycles were correlated to sensitive teeth at 72 h.

Resin infiltration treatment was found to be statistically significantly associated with differences in answers of aesthetic dissatisfaction between t0 and t1 (Wilcoxon signed-rank test, *p* = 0.0001) and those collected between t1 and t2 (Wilcoxon signed-rank test, *p* = 0.0078). Between t2 and t3 (Wilcoxon signed-rank test, *p* = 0.25) and between t3 and t4 (Wilcoxon signed-rank test, *p* = 0.99) no statistical differences were found in answers of patients about dissatisfaction.

Details of statistical analysis were shown in Table 3 and Table 4.

Smoking cigarettes did not affect the satisfaction of results, no statistical differences were highlighted between smokers and non-smokers patients at t0 and t1.

## 4. Discussion

Resin infiltration technique in treatment for dental fluorosis lesions was investigated and parameters linked to clinical evaluation and answers of patients were recorded and analyzed for a follow-up of 1 year, showing a satisfying resolution of lesions as well as encouraging answers about the viability of the treatment.

The manufacturer recommends to not exceeding with the etching cycles in order to avoid cavitated lesions of the enamel surface; however, in the present work high number of etching cycles were in some case performed without such side effects. Some clarification is therefore needed in this regard; etching cycles were found to be correlated to the dimension of lesions and TSIF. This seemed to indicate that larger lesions required a greater number of etching cycles to be solved. Surely, a limitation of this study was attributable to the assessment of dimension of lesions: authors measured extension of lesions in mm, however it would have been more predictable to evaluate lesions in terms of depth. The severity of lesion was, therefore, assessed by the clinician who decided how many etching cycles to perform, basing his evaluation on experience, the dimension of lesions and TSIF. However, literature reports that the width of lesion is not always responsible for severity, sometimes the marked borders are predictors for deeper lesions that those nuanced and opalescent [23]: this aspect was afterwards noticed in lesions treated, nevertheless, it was not reported as result because it is mostly related to impression of the clinician.

However, in vivo validated methods to perform this assessment are not tested any more, this led us to measure only width of lesions in our study [23,24].

Nonetheless, a correlation between etching cycles and dimension of lesions was found, this seems to point the attention on the possibility that larger lesions were not so deep, explaining the fact that cavitated lesions after the treatment were not highlighted.

With these highlights in mind, it could be assumed that etching cycles are correlated to TSIF confirming the previous issue. Furthermore, this could seem a critical point in terms of dental sensitivity whether deep lesions are treated, however, results are encouraging because a correlation was found only between etching cycles and pain during treatment but dental sensitivity after 72 h was not statistically significantly correlated to this parameter.

Several studies had investigated this issue [25,26], largely supports the hypothesis of viability to use resin infiltration to treat fluorotic lesions with satisfying results. Indeed, the stability of results obtained was attested overtime by the satisfaction of patients and by clinical assessment at each time-points.

Lesions were nearly not detectable at t1 as well as during all the observation period, this attests how lesions were completely solved and results were steady. In our opinion, well aware of the importance of spectrophotometer to properly evaluate all changes of color, the satisfaction of patients and evaluation of clinicians are not parameters to be underestimated. This technique aims, actually, to fill up hypomineralized porous enamel thanks to a resin with an RI close to that of healthy enamel (RI = 1.62), in order to mask the enamel defect [27]; with this in mind, aesthetical evaluation of result and stability of the effect is a good goal to reach. Surely, it might be really interesting to evaluate the real values of teeth’s color over time.

Regarding aesthetic dissatisfaction for white spots lesions, a significant statistical difference was noticed between values at t0 and t1, as well as for those between t1 and t2. This aspect is of great interest, it indicates that result obtained at the end of the treatment improved during the first three months of follow-up and patients answered more positively regarding the aesthetic results. This improvement was no longer highlighted after the third month of observation, likely meaning that the result of infiltration becomes stable and it is not subject to changes, at least about patient perception.

This is in agreement with recent studies, even if the sample size in literature are smaller [28,29].

An interesting aspect was the aesthetical perception of lesions at baseline (t0) and of results at t1 between smoker and non-smokers patients. No differences were highlighted among two groups, however, this does not necessarily mean that smoking cigarette did not affect results but rather that it not influence the perception of subjects about their aesthetical satisfaction. For this reason, the authors did not perform further statistical analysis to investigate the potential correlation between the number of smoked cigarettes and satisfaction.

In this perspective, it is a must to remember that an evaluation by spectrophotometer would have been more accurate and surely evidence-based: however, the aim of this study was to assess the satisfaction of patients in terms of aesthetic perception of resin infiltration treatment and not to have evidence-based data on the stability of color of lesions.

Dental fluorosis seemed to influence self-esteem and aesthetic perception of patients, especially young people and sometimes is accountable of dental sensitivity [30,31]. Patients treated in this work reported a Shiff Air Index Sensitive Scale of 0 in all cases, except that of a subject suffering for periodontal disease: in this case the index did not change before and after treatment confirming values reported among all sample.

Furthermore, it is reported how fluorosis lesions are positive correlated to incidence of dental caries in children [32]. This could be an issue to take into consideration in planning remineralization of white spots of fluorosis to prevent incidence of caries or worsening of lesions, nevertheless a recent review and meta-analysis highlights that regaining the surface hardness of resin infiltrated-treated white spots lesions similar to sound enamel is doubtful. However, same authors indicated that resin infiltration was evaluated more effective than other methods for enhancing surface hardness [33].

With this in mind, this technique has to be considered useful in treating both aesthetical side of the problem and that functional, thanks to viability in improving, even if not completely restoring, the hardness of enamel. Therefore, this opportunity surely gains an important position in the scenario of the treatment of white spots lesions, especially thanks to the viability to solve large lesions with steady results over time, avoiding side effects such as dental sensitivity of discomfort of patients.

Authors are well aware that the present study represents just a pilot research, whose the large sample and the long-lasting follow-up, compared to those in the present literature, are the strength points. Nevertheless, it could be improved and the same technique could be tested in two different groups or, moreover, the resin infiltration could be compared to other similar materials. With regard to this, the authors chose to design the study in this way to understand the features and the aspects of this technique that deserve to be more deeply investigated. Done this, it may be possible to design appropriate material and methods to perform further studies, based on these findings. Unfortunately, the recent literature does not provide sound results on a large sample of lesions and long-lasting follow-up. [28,29].

It might be of great interest to deeply study the correlation between features of lesions, especially that predictor for depth and the resulting cavitated lesions after treatment as well as to establish a validated method to assess the depth of lesions. In this way it could be possible to establish a more predictable and strict protocol to follow in support of more focused researches.

At this regard, our results showed a high satisfaction of patients regarding the duration of the treatment, meaning that, also in cases where several lesions were treated at the same time, the duration of the procedure was well accepted by all participants interviewed.

## 5. Conclusions

Resin infiltration technique seems to be a valuable option to solve non-aesthetic white spots due to fluorosis. Furthermore, it ensures results in stable over time on a large sample of lesion.

Additional studies, likely carried out by a spectrophotometer, could lead to more evidence-based results.

## Figures and Tables

**Table 1:** Demographic features of the sample.

Demographic Features	Number of Patients
**Gender**	
Male	16
Female	14
**Age-Groups (years)**	
<20	4
20–30	20
31–40	3
41–50	3
**Smokers**	
Yes	13
Not	17

**Table 2:** Aesthetic dissatisfaction at timepoints (Visual Analogue Scale—VAS).

t0	t1	t2	t3	t4
8	2	0	0	0
4	0	0	0	0
6	3	0	0	0
8	0	0	0	0
6	1	0	0	0
6	0	0	0	0
7	0	0	0	0
8	0	0	0	0
8	4	1	0	0
5	1	0	0	0
7	0	0	0	0
9	0	0	0	0
10	0	0	0	0
9	2	1	0	0
10	0	0	0	0
5	2	2	0	0
7	2	1	0	0
5	1	1	0	0
7	0	0	0	0
7	1	0	0	0
7	0	0	0	0
9	0	0	0	0
9	0	0	0	0
7	1	0	0	0
7	0	0	0	0
6	2	0	0	0
5	0	0	0	0
9	0	0	0	0
10	0	0	0	0
8	0	0	0	0

**Table 3:** Correlation between etching cycles and variables. Spearman’s correlation.

Variables	Spearman’s Coef	Significance (*p*)
Sensitive Teeth at 72 h	0.14	0.06
Pain During Treatment	0.21	0.01
Lesion Dimension at t0	0.24	0.001
Lesion Dimension at t1	0.45	0.00001
TSFI * at t0	0.57	0.00001
TSFI * at t1	0.46	0.00001

TSFI *: Tooth Surface Index of Fluorosis.

**Table 4:** Wilcoxon signed-rank test.

Aesthetic Dissatisfaction	*p* Values
t0–t1	0.0001
t1–t2	0.0078
t2–t3	0.25
t3–t4	0.99

## Data Availability

The data of this study are available from the corresponding author upon reasonable request.
